# Examining user fee reductions in public primary healthcare facilities in Kenya, 1997–2012: effects on the use and content of antenatal care

**DOI:** 10.1186/s12939-020-1150-8

**Published:** 2020-03-14

**Authors:** Mardieh L. Dennis, Lenka Benova, Catherine Goodman, Edwine Barasa, Timothy Abuya, Oona M. R. Campbell

**Affiliations:** 1 Faculty of Epidemiology & Population Health, London School of Hygiene & Tropical Medicine, London, UK; 2 Department of Public Health, Institute of Tropical Medicine, Antwerp, Belgium; 3 Faculty of Public Health & Policy, London School of Hygiene & Tropical Medicine, London, UK; 4 Health Economics Research Unit, KEMRI-Wellcome Trust Research Programme, Nairobi, Kenya; 5 Nuffield Department of Medicine, University of Oxford, Oxford, UK; 6 Population Council, Nairobi, Kenya

**Keywords:** Universal healthcare coverage, User fees, Antenatal care, Maternal health, Kenya

## Abstract

**Background:**

In 2004, The Kenyan government removed user fees in public dispensaries and health centers and replaced them with registration charges of 10 and 20 Kenyan shillings (2004 $US 0.13 and $0.25), respectively. This was termed the 10/20 policy. We examined the effect of this policy on the coverage, timing, source, and content of antenatal care (ANC), and the equity in these outcomes.

**Methods:**

Data from the 2003, 2008/9 and 2014 Kenya Demographic and Health Surveys were pooled to investigate women’s ANC care-seeking. We conducted an interrupted time series analysis to assess the impact of the 10/20 policy on the levels of and trends in coverage for 4+ ANC contacts among all women; early ANC initiation and use of public facility-based care among 1+ ANC users; and use of public primary care facilities and receipt of good content, or quality, of ANC among users of public facilities. All analyses were conducted at the population level and separately for women with higher and lower household wealth.

**Results:**

The policy had positive effects on use of 4+ ANC among both better-off and worse-off women. Among users of 1+ ANC, the 10/20 policy had positive effects on early ANC initiation at the population-level and among better-off women, but not among the worse-off. The policy was associated with reduced use of public facility-based ANC among better-off women. Among worse-off users of public facility-based ANC, the 10/20 policy was associated with reduced use of primary care facilities and increased content of ANC.

**Conclusions:**

This study highlights mixed findings on the impact of the 10/20 policy on ANC service-seeking and content of care. Given the reduced use of public facilities among the better-off and of primary care facilities among the worse-off, this research also brings into question the mechanisms through which the policy achieved any benefits and whether reducing user fees is sufficient for equitably increasing healthcare access.

## Background

In the decades since the widespread African independence movements of the mid-1900s, countries in sub-Saharan Africa have struggled to develop economically sustainable healthcare financing models that ensure universal coverage of essential health services. Faced with budgetary constraints and external pressures to both independently finance local healthcare systems and reduce government spending, many African countries introduced user fees in public sector health facilities in the late 1980s [1, 2]. Proponents of user fees argued that these charges would improve efficiency and the quality of health services by generating revenue to help cover costs for general operations and the supply and maintenance of health commodities and infrastructure [2]. Others argued that user fees were important for discouraging unwarranted use of care and ensuring that people attach value to health services [3].

In reality, as user fees were being introduced widely across African countries from the late 1980s to 1990s, emerging evidence during that same period raised doubts as to whether the expected benefits of user fees were always achieved in practice. For example, in settings such as Burkina Faso, the Gambia, Ghana, Kenya, Lesotho, Mozambique, Niger, Swaziland, Zaire, Zambia, and Zimbabwe, the introduction or increase of user fees was immediately followed by reduced care-seeking in public sector health facilities [4–13]. Also, contrary to expectations, available evidence at that time suggested that unwarranted health service use comprised a small proportion of the cases contributing to reduced service volumes [11]. Research from Kenya, Lesotho, and Swaziland further suggested that introducing or increasing fees in public facilities sometimes shifted patients away from the public sector and into the private sector, rather than decreasing overall demand [5, 6, 8]. Studies on health service cost recovery from several countries in Africa revealed that while user fees did generate revenue, often this was low and insufficient for making impactful investments in quality improvement [4, 6, 11, 14, 15]. Further, evidence from countries such as Ghana, Kenya, and Zimbabwe suggested that inefficient management of this revenue also inhibited user fees from translating into large improvements in quality of care [4, 10, 14].

Kenya, similarly to these other African countries, has struggled to develop a health financing system that sustainably and equitably increases access to good quality care while ensuring that its citizens have financial risk protection from the hardship that may result from out-of-pocket healthcare payments. Kenya’s public health system is organized into six levels ranging from community-based care (level 1) to tertiary hospitals (level 6) [16, 17]. Level 1 consists of health promotion and awareness-raising activities at the community level; levels 2–3 include primary health care facilities, including dispensaries and health centers; and levels 4–6 include county and national referral hospitals [16, 17].

**Fig. 1:**
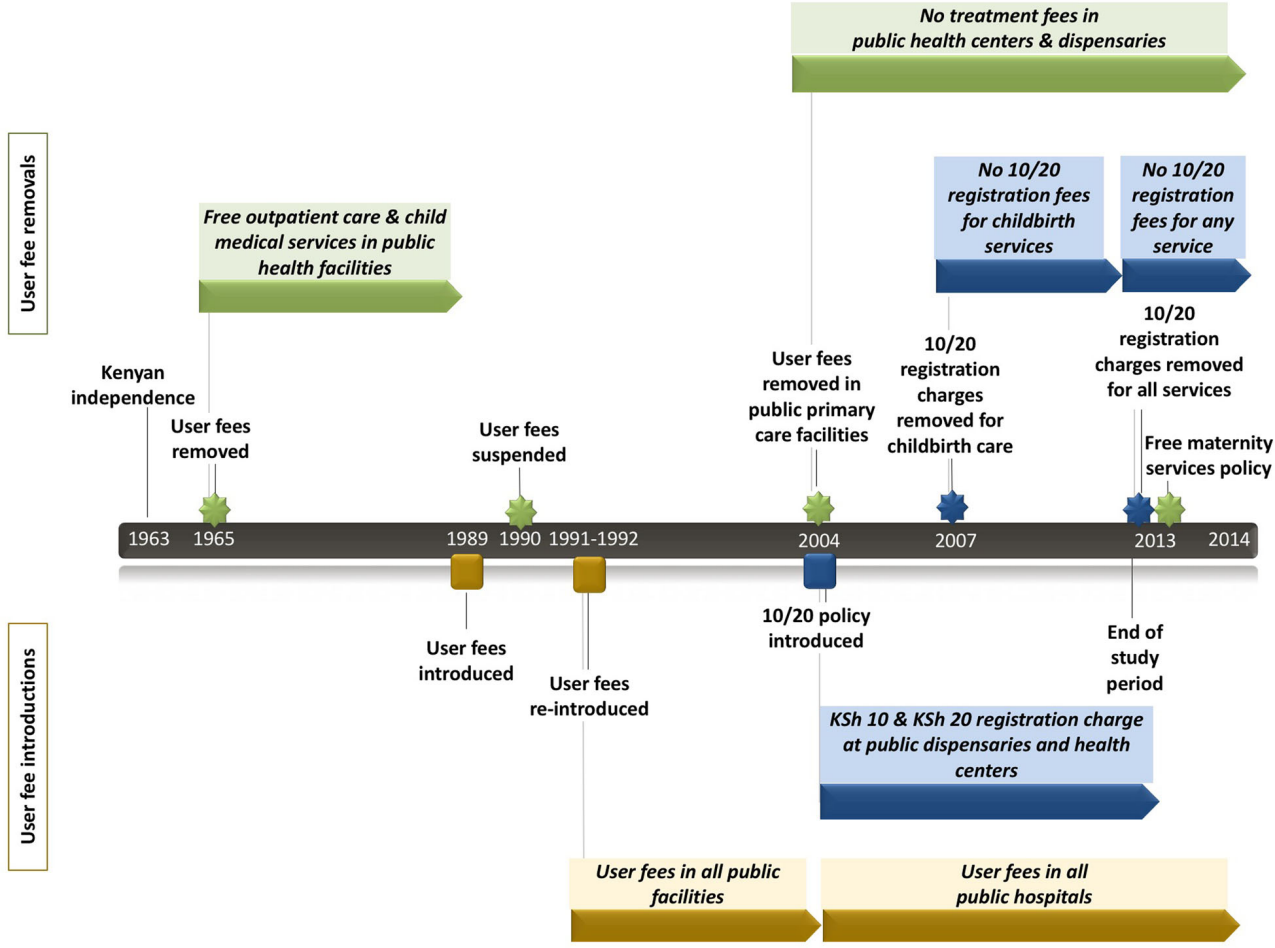
Timeline of public health facility user fee reforms in Kenya

Since introducing user charges in 1989 for the first time after independence, Kenya has implemented a series of user fee removals, re-introductions, and reductions, sometimes targeting specific levels of care (Fig. 1) [18, 19]. Although these user fees were introduced in conjunction with a waiver system for fee exemptions based on ability to pay, there were concerns about the negative impact of the user fees on access to health services among the poor. This led to fees being suspended in 1990 and subsequently re-introduced in phases between 1991 and 1992, with a stronger focus on ensuring that the user fee policy and fee waiver system were implemented properly [12, 18]. In 2003, the Kenyan government developed an economic recovery strategy that declared that investing in a healthy population, and in particular the poor, was a necessity for accelerating economic growth [20]. Within this context, Kenya’s Minister of Health in 2004 declared that user fees were to be eliminated in public primary healthcare facilities (health centers and dispensaries), effective 1 July 2004, and instead replaced with nominal registration charges of 10 Kenyan shillings (KSh) in dispensaries and KSh20 in health centers (2004 US$0.13 and 0.25). Under this 10/20 policy, certain groups and services were exempted from any payment, including the poor, children below 5 years, and those seeking treatment for malaria and tuberculosis [21]. While multiple reports indicate that pregnant women seeking antenatal care (ANC) were also intended to be exempted from any payment under the 10/20 policy, this may not have been implemented consistently in practice [22–24]. In 2007, the government also announced that women seeking facility-based childbirth care would be exempt from paying the 10/20 registration fees [21]. Most recently, in 2013, the Kenyan government removed user fees for all services provided in public health centers and dispensaries, and introduced free maternity services in public facilities at all levels from primary to tertiary [25], policies which both stand to this day.

While a few studies have examined the short-term impact of the 10/20 policy, there is little evidence of the long-term effects of the policy leading up to Kenya’s 2013 health sector financing reforms. An evaluation conducted shortly after the 10/20 policy was introduced in 2004 suggested that public health centers and dispensaries experienced a sharp increase in patient volumes in the months immediately following the policy change [21, 22]. The rate of increase in patient numbers eventually declined, but the patient volumes remained higher than those seen before the policy [21, 22]. A more recent study of the long-term population-level effects of the 10/20 policy on women’s source of childbirth care by Obare and colleagues found that the 2004 policy did not increase the proportion of women delivering in public sector facilities or the change in public facility deliveries over time; instead the policy was associated with an immediate increase in the proportion of poor women who delivered outside of a health facility [26]. Further, the study found that after the removal of 10/20 registration fees for childbirth care in public health centers and dispensaries in 2007, there was an immediate increase in the use of public facility-based childbirth care and decrease in non-facility births among the wealthiest women, but no change in childbirth service-seeking among the poorest women.

As the government of Kenya continues to develop their health financing mechanisms for maternal health, it is critical to understand the long-term effects of past reforms and identify strategies for ensuring that current and future financing policies have lasting impact. Given the strong link between ANC and subsequent use of intrapartum and postpartum maternal health services [27–34], it is important to investigate the relationship between the implementation of the 10/20 policy and women’s experiences during pregnancy, and whether this may help explain why the policy did not increase coverage of facility deliveries, particularly among the poor. Additionally, studying ANC allows us to examine the effect of the policy on multiple dimensions of service use beyond coverage, including number and timing of visits, type of provider, and content of care. The objective of this paper is therefore to examine if the introduction of the 2004 10/20 policy was associated with any changes in ANC care-seeking practices and quality of care, as measured by the content of ANC. Specifically, this study assesses if the removal of user fees and introduction of the 10/20 registration charge policy was associated with increases in frequency of ANC visits, early ANC initiation, and use of public sector ANC services. As the 10/20 policy specifically targeted public primary care facilities, we also examined whether there was a shift from secondary and tertiary healthcare facilities (hospitals) towards lower-level facilities among users of public sector care. Additionally, we investigated whether any such evidence of increased use of ANC services was accompanied by reduced content of care, resulting from higher demand on public health services. Lastly, as the policy was intended to ensure that the most vulnerable could access essential care, we explored whether any observed changes in service-seeking and content of care associated with the 10/20 policy were equitable between better-off and worse-off women.

## Methods

### Data and study population

We used the 2003, 2008/9 and 2014 Kenya Demographic and Health Survey (DHS) woman’s questionnaire datasets for this analysis. We excluded earlier surveys (1998, 1993, 1989), as they did not collect information on one or more of the study’s key outcomes of interest. The 2003 and 2008/9 datasets sampled a total of 8561 and 9057 households, respectively [35, 36]. The 2014 dataset sampled a total of 36,430 households; of these, one in every two households was randomly selected to complete a long version of the woman’s questionnaire, and the other half were administered a shorter woman’s questionnaire [37]. As the shorter questionnaire did not ask questions related to the source or content of ANC, we limited our analysis of the 2014 dataset to the 17,409 households in which women completed the full questionnaire.

All women aged 15–49 years in the included households were selected for participation in the surveys. Among the 31,380 eligible women interviewed across the three surveys, all 15,230 women who reported having their most recent live birth with an estimated date of conception before July 2012 were included in this analysis. We used women’s reports on their most recent live birth rather than all live births, as the included surveys only asked questions on ANC for women’s most recent births.

### Study outcomes

We examined one indicator of ANC coverage among all women in the analysis sample: 4+ ANC, defined as the proportion of women reporting four or more ANC contacts. We did not examine use of 1+ ANC, as this indicator remained above 90% throughout the study period [179,310,311]. We examined the proportion of women receiving 4+ ANC because at the start of the pregnancies included in this analysis (2012 and earlier), the World Health Organization (WHO) was still recommending that women should make a minimum of four ANC visits during pregnancy, though they subsequently increased to a minimum of eight visits [38].

Among users of 1+ ANC, we examined timing of ANC initiation and source of care. We defined early ANC as ANC users who had their first visit during the first 3 months of their pregnancy. For source of care, we categorized ANC users into two categories: any public sector facility-based ANC and no public sector facility-based ANC. We considered facilities owned by the government to be public and all other facilities, including for-profit, non-profit, and faith-based, to be private. As women could report receiving ANC from more than one location, we considered any public sector facility-based ANC to include (a) women who received ANC exclusively from a public health facility and (b) women who received care both in a public health facility as well as in a private facility or at home/other location. We categorized women who received care exclusively in a private facility and/or exclusively at home or another location as having received no public sector facility-based ANC.

Among users of public sector facility-based ANC, we investigated whether there were any changes in facility level and content of care. With regard to level of care, we examined the distribution of women who sought care in public primary care facilities (dispensaries or health centers) versus public secondary and tertiary facilities (hospitals). In terms of content of care, we examined six components of ANC routinely assessed in the DHS questionnaires: (1) blood pressure measured; (2) urine sample taken; (3) blood sample taken; (4) received tetanus injection; (5) given iron supplements; and (6) told about pregnancy complications, at least once during pregnancy [39]. We considered women who reported receiving all six of these components to have received good content of care. Although the 10/20 policy specifically targeted public primary care facilities, we were unable to examine the impact of the 10/20 policy on the content of care received by the subset of women who accessed care in public primary care facilities due to small sample sizes in some of the study periods (Additional file 1). We therefore examined content of care among all users of public facility-based ANC.

In addition to estimating the effects of the 10/20 policy on the key study outcomes, we conducted stratified analyses to examine whether any observed effects were equitable between women of different socioeconomic groups. We defined women’s socioeconomic status using wealth quintiles based on the household asset indices derived from the DHS household questionnaire [37]. For each of the ANC outcomes, we ran the analyses separately among women from the top two (40%) household wealth quintiles (better-off) and among women in the bottom three (60%) quintiles (worse-off). We included tables with the results stratified by urban and rural residence in Additional file 2.

### Statistical analysis

We conducted an interrupted time series analysis using segmented linear regression models to assess the impact of the introduction of the 10/20 policy in 2004 on the study outcomes. As this study aimed to examine whether the 10/20 policy influenced timing of ANC initiation, measured from the start of pregnancy, and subsequent use of ANC, we categorized each woman’s outcomes into a half-year period according to her estimated time of conception. To set up the data for analysis, we appended the three DHS datasets and estimated outcomes for each half-year from July 1997 to December 2012. Each half-year estimate was weighted to account for the multi-stage cluster sampling design of the DHS.

We assumed each birth had a gestational age of 38 weeks, based on a weighted median of the most recent estimates of the distribution of full term and preterm birth in sub-Saharan Africa [40, 41]. Additional file 3 contains our calculations for the weighted median gestational age. To approximate time of conception, we subtracted 38 weeks from the date of each woman’s most recent birth. Based on these calculations, approximately 2% of women included in the sample could potentially access ANC services both before and after the 10/20 policy was introduced, as their pregnancies spanned the half-year periods immediately before and after the policy change. Our analysis categorized women according to when their pregnancy began; thus, this 2% sub-sample was treated as if they received care before the policy change.

For each model, we tested for evidence of the impact of the 2004 10/20 policy introduction on the study outcomes. As there are too few data points after the introduction of the free maternity services policy in June 2013 to examine its impact, our analysis excludes births that were conceived in the half-years beginning July 2012 and later. We tested the data for autocorrelation using the Cumby-Huizinga test and identified evidence of serial autocorrelation in even number lags [42]. We assumed that this was due to seasonality, with observations from one half-year (e.g. January to June of year X) correlated with observations from two half-years prior (e.g. January to June of year X-1). We corrected for this using the Newey estimator with a lag of two [42]. For the purposes of this analysis, we considered the period from July 1997 until just before the policy change on 1 July 2004 to be “pre-policy,” (14 half-year periods) and the period from just after 1 July 2004 through December 2012 to be “post-policy” (17 half-year periods). As the estimates for each half-year period were derived from survey data and have different sample sizes and levels of uncertainty, we weighted our time series analysis by the inverse of the variance for the estimates at each half-year period. This means that time points with greater uncertainty around the estimate contributed less to model, while time points with lower uncertainty contributed more to the models. Additional file 1 contains a table listing the sample size for each study population by half-year. All analyses were conducted in Stata SE version 15.

For each outcome, we reported two measures of the impact of the 10/20 policy: the immediate change in level and the immediate change in slope. The immediate change in level estimates the amount by which the percent of the study population reporting a particular outcome changed immediately after the 10/20 policy was introduced. The immediate change in slope estimates the amount by which the change over time in the outcome sped up (accelerated) or slowed down (decelerated) immediately after the 10/20 policy was introduced.

In addition to these measures of the impact of the 10/20 policy, we also reported on three general estimates of the level and changes over time in the outcomes: the pre-policy starting level, the pre-policy half-yearly trend, and the post-policy half-yearly trend. The pre-policy starting level is a model-based estimate of the percentage of the study population reporting the outcome of interest during the first half-year period in the analysis. As this is a model-based estimate rather than a direct estimate, it was possible for the results to return a point estimate or confidence interval below 0 % or above 100%. In such cases, we truncated the estimates and confidence intervals to between zero to 100% to exclude impossible values. The pre-policy half-yearly trend estimates the average change over time in the level of the outcome between each six-month period from the first half-year in the analysis until the period immediately before the 10/20 policy change. Similarly, the post-policy half-yearly trend estimates the average change over time in the level of the outcome between each six-month period after the 10/20 policy. Both of these measures refer to the general trends over time, rather than the effect of the 10/20 policy on these trends.

We also displayed the outcome measures graphically in Additional file 4. In the graphs, the x-axis represents half-year periods. For example, “h1” represents the first half of the year (January–June) and “h2” represents the second half of the year (July–December). The lines represent the predicted trend over time in coverage of the outcome variable. The circles represent the estimated coverage during a given half-year. The size of each circle is proportional to the inverse of the variance for the estimated coverage during that half-year period.

## Results

### Number of ANC visits (4+ ANC)

In contrast to the consistently high percentage of women making at least one ANC visit during pregnancy, only 62.3% of women made the recommended minimum of four ANC visits during pregnancy at the beginning of the study period (Table 1). The results show that before the introduction of the 10/20 policy, the proportion of pregnant women who made 4+ ANC contacts decreased by approximately 1.2 percentage points every 6 months (*p* = 0.009). After the 10/20 policy was introduced, the trend in use of 4+ ANC accelerated by 2.4 percentage points per half-year (*p* = 0.001); however, there was no immediate change in the proportion of women who made at least four ANC visits. Use of 4+ ANC increased by 1.1 percentage points per half-year (*p* = 0.003) after the 10/20 policy was introduced.

**Table 1:** Use of 4+ ANC among most recent births

	4+ ANC (All women)		4+ ANC (Worse-off women)		4+ ANC (Better-off women)	
	Estimate [95% CI]	***p***-value	Estimate [95% CI]	***p***-value	Estimate [95% CI]	***p***-value
Pre-policy starting level	62.3% [57.4,67.1%]		51.6% [47.0,56.3%]		78.9% [72.1,85.8%]	
Pre-policy half-yearly trend	−1.2% [− 2.2,-0.3%]	0.009	− 0.8% [− 1.5,-0.1%]	0.033	− 2.0% [−3.2,-0.9%]	0.001
Immediate change in level	+ 0.3% [− 11.8,12.3%]	0.965	−5.8% [− 18.9,7.3%]	0.372	+ 10.4% [0.0, 20.7%]	0.051
Immediate change in slope	+ 2.4% [1.1,3.6%]	0.001	+ 2.0% [0.9,3.1%]	0.001	+ 2.9% [1.5,4.2%]	< 0.001
Post-policy half-yearly trend	+ 1.1% [0.4,1.8%]	0.003	+ 1.2% [0.4,2.1%]	0.006	+ 0.8% [0.2,1.4%]	0.010

At the start of the study period, an estimated 51.6% of worse-off women and 78.9% of better-off women made a minimum of four ANC visits. Before the 10/20 policy was introduced, use of 4+ ANC significantly decreased over time among both worse-off and better-off women. Although the proportion of better-off women making 4+ ANC contacts may have increased by 10.4 percentage points immediately after the 10/20 policy was introduced (*p* = 0.051), there was no evidence of an immediate impact on the level of 4+ ANC use among worse-off women. The 10/20 policy was associated with 2.0 (*p* = 0.001) and 2.9 (*p* < 0.001) percentage points per half-year accelerations of the trends in 4+ ANC use among worse-off and better-off women, respectively. Thus, after the 10/20 policy was introduced, use of 4+ ANC increased by 1.2 percentage points per half-year (*p* = 0.006) among worse-off women and 0.8 percentage points per half-year (*p* = 0.010) among better-off women.

### Timing of ANC initiation among users of 1+ ANC

At the start of the study period, only 14.0% of 1+ ANC users reported making their first ANC visit within the first 3 months of their pregnancy (early ANC initiation) (Table 2). Prior to the introduction of the 10/20 policy, early ANC initiation remained constant over time. While there was no immediate change in the percentage of women who started ANC early after the policy was introduced, the trend in early ANC initiation accelerated by 1.0 percentage points per half-year (*p* = 0.008) after the policy change. After the introduction of the 10/20 policy, the proportion of 1+ ANC users who initiated ANC early increased by 0.7 percentage points every 6 months (*p* < 0.001).

**Table 2:** Early ANC initiation among users of 1+ ANC

	Early ANC (All women)		Early ANC (Worse-off women)		Early ANC (Better-off women)	
	Estimate [95% CI]	***p***-value	Estimate [95% CI]	***p***-value	Estimate [95% CI]	***p***-value
Pre-policy starting level	14.0% [10.2,17.9%]		4.5% [0.0,9.5%]		20.4% [15.1,25.6%]	
Pre-policy half-yearly trend	−0.3% [− 0.9,0.3%]	0.355	+ 0.7% [0.0,1.3%]	0.047	−1.0% [− 2.0,0.0%]	0.049
Immediate change in level	+ 2.6% [− 2.5,7.6%]	0.303	−4.5% [− 11.4,2.4%]	0.191	+ 9.3% [− 0.4,18.9%]	0.059
Immediate change in slope	+ 1.0% [0.3,1.7%]	0.008	−0.2% [− 0.9,0.5%]	0.609	+ 2.0% [0.8,3.3%]	0.002
Post-policy half-yearly trend	+ 0.7% [0.5,0.9%]	< 0.001	+ 0.5% [0.2,0.8%]	0.006	+ 1.0% [0.5,1.5%]	< 0.001

At the start of the study period, 20.4% of better-off ANC users started ANC within the first 3 months of pregnancy, while coverage of early ANC initiation was 4.5% among worse-off ANC users. Prior to the policy change, early ANC initiation increased by 0.7 percentage points per half-year among worse-off ANC users (*p* = 0.047) and decreased by 1.0 percentage point per half year (*p* = 0.049) among better-off ANC users. Among better-off ANC users, the trend in early ANC accelerated by 2.0 percentage points per half-year (*p* = 0.002) immediately after the 10/20 policy was introduced. There was no immediate change in the level of or trend in early initiation among worse-off ANC users. In both groups, early ANC initiation gradually increased over time during the years after the 10/20 policy was introduced.

### Source of care among users of 1+ ANC

An estimated 66.0% of 1+ ANC users received care from a public sector health facility at the start of the study period in 1997 (Table 3). Use of public health facility-based ANC increased by 1.0 percentage points every 6 months before the 10/20 policy was introduced (*p* = 0.044); however, the policy was not associated with any immediate change in the percentage of 1+ ANC users who sought care from a public facility. The results indicate that the 10/20 policy did not appear to accelerate the previously increasing trend in use of public sector health facilities; instead, they suggest that the policy decelerated the trend in use of public health facilities by 1.0 percentage points per half-year (*p* = 0.042). After the 10/20 policy was introduced, use of public facility-based ANC remained constant over time.

**Table 3:** Use of ANC from a public sector health facility among users of 1+ ANC

	Any public facility (All women)		Any public facility (Worse-off women)		Any public facility (Better-off women)	
	Estimate [95% CI]	***p***-value	Estimate [95% CI]	***p***-value	Estimate [95% CI]	***p***-value
Pre-policy starting level	66.0% [59.6,72.4%]		69.2% [59.2,79.3%]		62.7% [58.2,67.2%]	
Pre-policy half-yearly trend	+ 1.0% [0.0,2.0%]	0.044	+ 0.7% [−0.8,2.1%]	0.358	+ 1.5% [0.7,2.2%]	< 0.001
Immediate change in level	+ 3.0% [− 6.1,12.1%]	0.499	+ 8.7% [−4.0,21.3%]	0.171	−5.0% [− 13.2,3.3%]	0.226
Immediate change in slope	−1.0% [−2.0,0.0%]	0.042	−0.4% [− 1.9,1.0%]	0.541	−1.7% [− 2.6,-0.9%]	< 0.001
Post-policy half-yearly trend	0.0% [− 0.2,0.2%]	0.976	+ 0.2% [0.0,0.4%]	0.041	− 0.3% [− 0.7,0.1%]	0.187

At the start of the study period, approximately 69.2% of worse-off women and 62.7% of better-off women received their ANC from a public sector health facility. Before the 10/20 policy was introduced, use of public facility-based ANC increased by 1.5 percentage points per half-year among better-off ANC users (*p* < 0.001), but remained constant over time among the worse-off. While the policy had no impact on the level of public facility-based ANC use among either group nor on the trend in use of public ANC services among the worse-off, the results suggest that the change over time in use of public facilities among better-off ANC users decelerated by 1.7 percentage points per half-year immediately after the policy change (p < 0.001). In the years after the 10/20 was introduced, use of public facility-based ANC increased by 0.2 percentage points per half-year (*p* = 0.041) among the worse-off.

### Use of primary care facilities among users of public facility ANC

Approximately 64.5% of all public facility ANC users received care from a primary care facility (dispensary or health center) at the beginning of the study period (Table 4). Use of primary care facilities remained constant over time both before and after the 10/20 policy was introduced, and the policy did not have any measurable impact on the use of primary care facilities among public facility-based ANC users.

**Table 4:** Use of primary care facilities among users of any public facility-based ANC

	Primary care facility (All women)		Primary care facility (Worse-off women)		Primary care facility (Better-off women)	
	Estimate [95% CI]	***p***-value	Estimate [95% CI]	***p***-value	Estimate [95% CI]	***p***-value
Pre-policy starting level	64.5% [59.2,69.8%]		65.9% [59.0,72.9%]		63.0% [57.3,68.8%]	
Pre-policy half-yearly trend	+ 0.4% [− 0.5,1.4%]	0.356	+ 1.2% [0.3,2.2%]	0.010	−0.7% [− 1.6,0.1%]	0.095
Immediate change in level	− 4.7% [− 14.6, 5.2%]	0.335	−9.5% [− 17.6,-1.4%]	0.023	0.3% [− 9.5,10.2%]	0.945
Immediate change in slope	− 0.7% [− 1.7,0.4%]	0.193	−1.3% [− 2.3,-0.3%]	0.013	+ 0.3% [− 0.9,1.4%]	0.653
Post-policy half-yearly trend	−0.2% [− 0.7,0.2%]	0.247	0.0% [− 0.4,0.3%]	0.803	−0.5% [− 1.1,0.2%]	0.164

An estimated 65.9 and 63.0% of worse-off and better-off public facility-based ANC users sought care from a primary care facility at the start of the study period, respectively. Before the 10/20 policy was introduced, use of primary care facilities increased by 1.2 percentage points every 6 months (*p* = 0.010) among worse-off women and remained constant over time among better-off women. The share of worse-off public facility users who sought care from a primary care facility decreased by 9.5 percentage points (*p* = 0.023) immediately after the 10/20 policy was introduced and use of primary care facilities decelerated by 1.3 percentage points per half-year (*p* = 0.013). Among the better-off, on the other hand, the 10/20 policy was not associated with any immediate effects the level of or change over time in primary care facility use. During the period after the 10/20 policy was introduced, use of primary care facilities remained constant over time among both worse-off and better-off public facility users.

### Content of care among users of public facility-based ANC

Only 9.4% of public health facility-based ANC users reported receiving all six routinely measured ANC components (good content of care), at the beginning of the study period in 1997 (Table 5). The results suggest that the percentage of public facility-based ANC users who received good content of ANC remained constant over time before the 10/20 policy was introduced, and the policy did not have any immediate effect on the level of coverage or change over time in receipt of good content of care. The proportion of public facility-based ANC users who received good content of care increased by 1.5 percentage points per half-year (*p* < 0.001) in the years after the 10/20 policy was introduced. In Additional file 5, we included tables with estimates of the proportion of women who received each of the six components as well as all six components combined, stratified by source of care and number of ANC contacts.

**Table 5:** Received good content of care among users of public facility-based ANC

	Received all 6 routine ANC components (All women)		Received all 6 routine ANC components (Worse-off women)		Received all 6 routine ANC components (Better-off women)	
	Estimate [95% CI]	***p***-value	Estimate [95% CI]	***p***-value	Estimate [95% CI]	***p***-value
Pre-policy starting level	9.4% [4.7,14.2%]		9.0% [4.6,13.3%]		9.9% [3.9,15.9%]	
Pre-policy half-yearly trend	+ 0.4% [−0.6,1.4%]	0.402	+ 0.1% [− 0.6,0.8%]	0.740	+ 0.9% [− 0.5,2.3%]	0.204
Immediate change in level	+ 4.9% [− 4.9,14.6%]	0.313	+ 3.6% [− 4.1,11.3%]	0.344	+ 6.5% [− 6.9,19.9%]	0.329
Immediate change in slope	+ 1.1% [− 0.2,2.3%]	0.087	+ 1.2% [0.3,2.2%]	0.014	+ 0.7% [− 0.9,2.4%]	0.356
Post-policy half-yearly trend	+ 1.5% [1.0,2.0%]	< 0.001	+ 1.3% [0.8,1.9%]	< 0.001	+ 1.6% [1.1,2.2%]	< 0.001

At the start of the study period, only 9.0 and 9.9% of worse-off and better-off public facility ANC users reported receiving good content of care, respectively. The proportion of women receiving good content of care remained constant over time prior to the policy change among both groups, and there was no immediate change in the proportion of women who received good content of care in either group. After the 10/20 policy was introduced, the rate of change in coverage of good content of ANC accelerated by 1.2 percentage points per half-year (*p* = 0.014) among worse-off public facility-based ANC users only. The proportion of women who received good content of care increased over time among both groups after the 10/20 policy was introduced.

Table 6 contains a summary of the impact of the 10/20 policy on all of the ANC outcomes examined among all women and stratified by wealth group. Additional file 2 includes similar tables illustrating greater positive impacts of the 10/20 policy among women living in urban areas compared to those in rural areas.

**Table 6:** Summary of the effects of the 10/20 policy on ANC

	Immediate change in level	Immediate change in slope
**(1) 4+ ANC (most recent births)**		
All women	none	increased
Worse-off women	none	increased
Better-off women	none	increased
**(2) Early ANC (users of 1+ ANC)**		
All women	none	increased
Worse-off women	none	none
Better-off women	none	increased
**(3) Public facility-based ANC (users of 1+ ANC)**		
All women	none	decreased
Worse-off women	none	none
Better-off women	none	decreased
**(4) Primary care (users of any public facility-based care)**		
All women	none	none
Worse-off women	decreased	decreased
Better-off women	none	none
**(5) Received good content of ANC (users of any public facility-based care)**		
All women	none	none
Worse-off women	none	increased
Better-off women	none	none

increased: increasing effect or trend, *p* < 0.05

decreased: decreasing effect or trend, *p* < 0.05

none: no effect, *p* > 0.05

## Discussion

### Summary of findings

Our study shows that over the past two decades, content of ANC has been universally low and there have been historical wealth-based disparities in the frequency and timing of ANC. The 10/20 policy was associated with the acceleration of the changes over time in use of 4+ ANC and early ANC initiation. The evidence suggests that the 10/20 policy was not associated with population-level increases in use of public facility-based ANC among ANC users nor on use of primary care facilities and content of care among users of public facilities. When disaggregated by wealth groups, the findings further suggest that the 10/20 policy may have been more beneficial to better-off women compared to poorer women.

### Understanding the causal mechanisms driving the 10/20 policy’s impact on ANC

Examining the findings stratified by wealth group raises important questions with regard to the causal mechanisms by which the 10/20 policy might have impacted the coverage, timing, frequency, and source of antenatal care. We hypothesized that reducing the cost of accessing ANC might lead to earlier ANC initiation, higher coverage of ANC, and increased number of ANC visits. Additionally, we expected that any increases in 4+ ANC coverage would be accompanied by increases in the proportion of ANC users who sought care from the public sector and the proportion of public facility-based ANC users who sought care at a primary care facility. Finally, we hypothesized that increased patient volumes in public primary care facilities as a result of the 10/20 policy might contribute to reduced content of care in the public sector.

Instead, we found that while the 10/20 policy had no impact on the timing of ANC initiation among worse-off women, the proportion of worse-off ANC users who made four or more ANC contacts began to increase at a faster rate immediately after the 10/20 policy was introduced. This suggests that for worse-off women, the policy was unable to immediately change practices around the timing of the first ANC visit among users, but successfully increased the proportion of women who made four or more ANC visits. We also found that while the policy did not increase the proportion of worse-off women using public sector care, it did accelerate improvements in receipt of good content of care among worse-off users of public facility-based ANC. As the policy change was associated with a shift towards greater use of public hospitals among worse-off users of public facility-based care, these findings suggest that the observed improvements in content of ANC among worse-off women may have been due to a combination of decreased use of public sector primary care facilities and increased number of ANC visits. Among better-off women, the 10/20 policy was associated with improvements in the timing, and number of visits. However, in contrast with our hypotheses, these improvements were also accompanied by decreased use of public sector facilities and no change in the use of primary care or content of care among users of public facility-based ANC.

A critical look into the design, implementation, and context of the 10/20 policy provides helpful insights for understanding why the policy may not have had the expected effect on a primary care service such as ANC. For instance, the 10/20 policy aimed to improve the financial accessibility of primary care but did not include any interventions to address other barriers that influence whether a woman accesses one or more ANC visits during her pregnancy. Although indirect financial costs, such as paying for transportation to and from health facilities, can serve as a significant barrier to care, the 10/20 policy only addressed direct costs for ANC in public primary care facilities. A study on catastrophic health spending in Kenya found that transportation costs account for nearly one quarter of households’ total out-of-pocket spending on health, and that the burden of transportation costs relative to total spending was highest among the poor [43]. This suggests that the high costs of transportation may have significantly influenced the impact of the 10/20 policy on ANC service use. In terms of non-financial barriers, a qualitative study on women’s beliefs and practices around ANC in Kenya revealed that while raising money for out-of-pocket fees sometimes required women to postpone their first ANC visit, factors related to women’s knowledge, beliefs, and traditions appeared to be more influential contributors to delayed ANC initiation [44]. Additionally, findings from two quantitative studies on determinants of ANC timing in Kenya also suggest that barriers including distance, knowledge, and customs might also inhibit early ANC initiation, as evidenced by the impact of factors such as living in a community with access to a community health worker, being from certain ethnic groups, parity, and being married on the timing of women’s first ANC visits [45, 46]. The fact that only better-off women experienced immediate increases in early ANC initiation after the introduction of the 10/20 policy therefore supports findings from other research suggesting that sometimes the impacts of user fee exemptions are inequitable because the poor tend to be disproportionally affected by indirect financial and non-financial barriers to healthcare [47].

With regard to source of care, there are many possible reasons why the policy did not lead to an increased use of public primary care facilities for ANC among the worse-off. For instance, although ANC services were intended to be available at the lowest levels of care, the 2004 Kenya Service Provision Assessment (KSPA) reported that only 77% of dispensaries offered ANC, compared to 86% of health centers and 84% of hospitals [16]. Further, the 2004 KSPA found that among facilities offering ANC, availability of the resources and infrastructure necessary for quality ANC was low, particularly in health centers and dispensaries [16]. In addition to this lower availability of quality ANC services in public primary care facilities, distrust related to the lack of clarity around the conditions of the policy; facilities’ failure to comply with the policy’s recommended fees; and concerns about the policy’s impact on quality of care may have also acted as deterrents. A qualitative study examining perceptions of the 10/20 policy among community members and health workers found that both the general public and health workers were confused about which aspects of care were covered under the policy and which services and groups were eligible for fee exemptions [21]. The study also found that some health providers and community members believed that the 10/20 policy reduced the cost of seeking care at the expense of quality of care, particularly in terms of drug availability [21]. Additionally, two nationally representative surveys of health facilities in Kenya found that 6 to 8 years after the 10/20 policy was introduced, health facility staff reported routinely overcharging for ANC in both health centers and dispensaries [23, 24]. An assessment conducted in 2012, for instance, found that public health centers and dispensaries reported charging KSh 58 and KSh 46 per ANC visit, respectively, while hospitals reported charging similar fees of KSh 55 per visit [24]. Finally, although the 10/20 policy purportedly reduced user fees in public primary care facilities, by many accounts, services were already being provided for free in some public dispensaries prior to the policy change [8, 11, 12, 21]. Thus, in some areas, rather than decreasing fees at the dispensary-level, the 10/20 policy potentially introduced official fees that previously did not exist.

The decreased use of public sector care among better-off ANC users after the 10/20 policy could be due to the comparative costs of seeking care in public versus private facilities after the policy change. A nationally representative survey of the fees charged by health facilities years after the 10/20 policy was introduced revealed that the cost of ANC was comparable between public and private facilities at the dispensary level [24]. Although the study also found that the fees for ANC in hospitals and health centers were higher in the private sector than in the public sector, the difference in pricing may not have been a sufficient barrier to stop better-off women from switching to private sector care [21, 24].

With regard to receipt of good content of ANC, the observed improvement in content of ANC among worse-off women may also be related to changes in the global guidelines on ANC around the same time that the 10/20 policy was introduced. From 1996 to 1998, the WHO conducted a multi-country randomized control trial of a new four-visit model of ANC delivery. Later, in 2002, the WHO published guidelines on the focused, or four-visit, ANC model and which interventions should be provided during each visit [48]. Simultaneously in 2001, this model was piloted in two out of Kenya’s then 72 districts and later scaled up to 19 additional districts in 2002 [49]. Although there were no national standards or guidelines for implementing focused ANC in Kenya at the time of the 10/20 policy change [49], it is plausible that as these guidelines were being piloted in select districts, there was a more general emphasis on improving the content of ANC throughout the country.

### Comparing effects of 10/20 policy on coverage of ANC vs. delivery care

Despite evidence that women’s experiences during ANC can influence care seeking for childbirth [27–34], most studies on the effects of user fees on maternal health service coverage have looked exclusively at delivery care. Our study demonstrates the value of examining the influence of health financing strategies on a broader range of maternal health outcomes and comparing findings across service types and sub-populations. The findings suggest that there were important differences and similarities between the impact of the 10/20 policy on coverage of antenatal care versus delivery care. In a recent paper using Kenya DHS data to examine the impact of the 2004 10/20 policy on coverage and source of delivery care, Obare et al. found that the proportion of women who delivered outside of a health facility immediately increased at the population level and among poor women (defined as the bottom two wealth quintiles), but had no immediate effect on home-based delivery care among wealthy women (defined as the top two wealth quintiles) [26]. Further, the study found no immediate effect of the 2004 10/20 policy on use of public facility-based delivery care; instead, the observed reduction in facility-based care was due to decreased use of private facilities and increased home-based births among the poor [26]. While Obare and colleagues’ findings suggest that the 2004 10/20 policy change was associated with decreased coverage of institutional deliveries, particularly among the poor, our findings suggest that the policy change was associated with increased coverage of 4+ ANC, particularly among the better-off. Thus, although the 10/20 policy’s impact on antenatal and delivery care coverage may have differed, both studies suggest that the policy contributed to better improvements in service coverage for women with higher socioeconomic status compared to those with lower socioeconomic status. These findings are consistent with other studies reporting that fee exemption policies may not always reduce inequities in access to care, particularly if non-financial barriers are not sufficiently addressed [47, 50–52].

There are a few plausible explanations for why the impact of the 10/20 policy change in 2004 might have differed between ANC and delivery care. For example, the impact of the policy might be related to the nature of the service. While ANC is an outpatient, largely preventative and promotive service, facility-based childbirth care is an inpatient service requiring a skilled provider. As a result, the proportion of health centers and dispensaries that offered delivery care in the early months after the policy change was substantially smaller than the proportion that offered ANC [16]. Due to these differences in service availability, the potential for the 10/20 policy to facilitate a population-level increase in use of facility-based delivery care was lower than for facility-based ANC. Secondly, it is likely that facilities’ inconsistent compliance with the policy impacted ANC and delivery care differently. Qualitative research conducted after the 10/20 policy was introduced suggests that health facilities often did not adhere to the policy’s recommended charges, and health care users were charged additional fees for certain drugs, laboratory tests, and services [21, 23, 24]. Health centers providing any inpatient services, in particular, reported that the 10/20 registration fees did not provide adequate cost recovery, which contributed to their noncompliance with the policy [21, 24]. Additionally, a nationally representative survey of Kenyan health facilities conducted in 2010 found that facility in-charges reported higher levels of overcharging for delivery services compared to ANC [23]. This study was conducted 6 years after the 10/20 policy was introduced and the findings may therefore be related to the duration of time passed since the policy change. However, given the comparatively higher costs for providing delivery care, it is conceivable that this practice of greater overcharging for delivery care was also prevalent during the time immediately after the policy change.

### Limitations

This study has some limitations. First, the data are subject to recall bias, as the DHS asks women to provide details about the antenatal care that they received for pregnancies that occurred up to 5 years prior to the interview date. Secondly, this analysis relies on categorizing women’s pregnancies by their estimated dates of conception. As it is difficult to accurately estimate the duration of a woman’s pregnancy using information on her child’s birthdate alone, our assumptions may have resulted in the misclassification of some births into the wrong half-year period. There was also potential for women who conceived just before the policy change to be pregnant both before and after its implementation. Such cases, though relatively few (approximately 2% of the study sample), could potentially have contributed to a crossover effect, whereby the impact of the policy on ANC may have been underestimated due to women who were categorized as conceiving before the policy change having access to its benefits. Measurement of the policy impact may have also been affected by small sample sizes in certain periods (Additional file 1); however, we adjusted for this by weighting each half-year observation by the precision of the outcome’s estimate for that period. Additionally, because the 10/20 policy was implemented at the national level, it was not possible to compare the time trends in a comparable control group that was not exposed to the policy change. Finally, although we used the content of antenatal care as a proxy for quality of care, this is not a comprehensive measure of quality of care, as it only measured a relatively small number of ANC components and did not assess more systems-level aspects of service quality or aspects related to respectful care.

## Conclusions

This study showed that the user fee reductions under the 10/20 policy in Kenya were associated with increased coverage and frequency of antenatal care. However, these improvements were not achieved through greater use of the public primary care facilities targeted under the policy, but instead through greater use of higher-level public facilities among the worse-off and private facilities among the better-off, leaving unanswered questions about the mechanisms through which the policy change may have affected service use patterns. Findings like these highlight the need to conduct qualitative research alongside the introduction of new health financing policies to better understand how they work in practice and the reasons for certain health seeking practices. This study also revealed that improvements in the timing and frequency of ANC were inequitable between better-off and worse-off women. On one hand, these findings imply that the policy may have increased out-of-pocket expenditures for the poor by pushing worse-off ANC users towards higher-level public-sector care for services that could be provided for lower costs in primary care facilities that complied with the 10/20 policy. On the other hand, the findings indicate that the policy may have stimulated more effective market segmentation by pushing the better-off towards the private sector and potentially increasing the public-sector resources available to those with lower ability to pay. Taken together, these findings contribute to the evidence that reducing user fees alone is not sufficient for equitably increasing access to primary healthcare services such as antenatal care. To ensure the success of the national health financing strategy that is currently being finalized in Kenya, policymakers must therefore develop strategies for concurrently addressing the key financial and non-financial barriers to recommended service-seeking practices.

## Supplementary information

**Additional file 1.** Sample sizes for time series analysis.

**Additional file 2.** Results from time series analysis stratified by residence.

**Additional file 3.** Mean gestational age calculations.

**Additional file 4.** Graphs of trends in ANC frequency, timing, source of care, and content of care.

**Additional file 5.** Receipt of individual ANC components.

## Data Availability

The datasets are available online upon request: http://dhsprogram.com/.
