# Supplementary material for: Examining user fee reductions in public primary healthcare facilities in Kenya, 1997–2012: effects on the use and content of antenatal care
Source: Int J Equity Health. 2020 Mar 14;19:35. doi: 10.1186/s12939-020-1150-8 (PMC7073011; doi:10.1186/s12939-020-1150-8)
Supplement: Supplementary file 1 — Additional file 1. Sample sizes for time series analysis. [file 12939_2020_1150_MOESM1_ESM.pdf]

APPENDIX 1: STUDY SAMPLE SIZES

	TOTAL SAMPLE				NON-WEALTHY SAMPLE				WEALTHY SAMPLE			
Half-year	All most recent births, weighted (N=14,901)	1+ ANC users, weighted (N=13,962)	Public facility ANC users, weighted (N=11,183)	Public primary care facility ANC users, weighted (N=7,239)	All most recent births, weighted (N=8,793)	1+ ANC users, weighted (N=8,065)	Public facility ANC users, weighted (N=6,771)	Public primary care facility ANC users, weighted (N=4,986)	All most recent births, weighted (N=6,108)	1+ ANC users, weighted (N=5,897)	Public facility ANC users, weighted (N=4412)	Public primary care facility ANC users, weighted (N=2,253)
1997h2	90	83	53	34	46	40	29	23	44	42	24	11
1998h1	178	161	113	70	98	82	58	35	79	79	54	36
1998h2	227	213	163	105	121	109	92	62	106	103	71	43
1999h1	259	230	153	89	145	126	84	53	114	105	69	36
1999h2	317	291	214	143	181	159	116	74	137	132	98	69
2000h1	362	336	216	146	222	201	122	91	141	135	93	56
2000h2	468	427	303	204	296	266	196	143	171	160	108	61
2001h1	578	518	376	261	367	316	227	178	210	201	148	83
2001h2	664	598	422	304	430	378	275	218	234	220	147	86
2002h1	642	561	390	275	410	353	236	186	232	208	154	89
2002h2	267	239	181	125	159	136	102	76	108	103	78	49
2003h1	65	59	51	21	22	19	15	8	43	40	35	12
2003h2	176	160	136	73	74	69	60	42	101	92	77	32
2004h1	216	198	167	121	108	99	84	69	108	100	83	52
2004h2	251	231	186	123	130	114	100	71	121	117	86	52
2005h1	301	287	236	157	158	147	128	96	143	140	108	61
2005h2	456	421	351	247	290	271	239	180	166	150	112	68
2006h1	448	424	336	226	270	251	215	165	178	173	122	60
2006h2	547	506	414	266	324	295	244	190	222	211	170	76
2007h1	575	534	443	263	360	324	283	197	215	210	160	67
2007h2	613	562	473	322	400	363	318	237	213	199	154	85
2008h1	326	298	251	175	212	187	163	123	115	110	88	52
2008h2	118	116	108	52	61	59	58	35	57	57	50	17
2009h1	365	341	290	153	164	151	135	85	201	190	155	69
2009h2	467	457	363	210	219	212	193	131	248	245	170	79
2010h1	507	492	401	239	269	257	222	165	238	235	178	74
2010h2	620	600	486	302	331	315	281	205	289	285	205	97
2011h1	664	645	533	342	373	357	319	225	291	288	214	117
2011h2	802	774	625	406	465	443	402	302	336	332	223	104
2012h1	848	826	695	449	507	486	435	327	341	340	260	122
