# Supplementary material for: Examining user fee reductions in public primary healthcare facilities in Kenya, 1997–2012: effects on the use and content of antenatal care
Source: Int J Equity Health. 2020 Mar 14;19:35. doi: 10.1186/s12939-020-1150-8 (PMC7073011; doi:10.1186/s12939-020-1150-8)
Supplement: Supplementary file 2 — Additional file 2. Results from time series analysis stratified by residence. [file 12939_2020_1150_MOESM2_ESM.pdf]

APPENDIX 2: Study outcomes stratified by rural/urban residence

Table A2.1 Use of 4+ ANC among most recent births

	4+ ANC (all women)		4+ ANC (rural)		4+ ANC (urban)	
	Estimate [95% CI]	p-value	Estimate [95% CI]	p-value	Estimate [95% CI]	p-value
Pre-policy starting level	62.3% [57.4%,67.1%]		56.1% [50.1%,62.1%]		84.0% [78.7%,89.2%]	
Pre-policy half-yearly trend	-1.2% [-2.2%,-0.3%]	0.009	-0.9% [-1.9%,0.2%]	0.096	-2.3% [-3.1%,-1.6%]	<0.001
Immediate change in level	+0.3% [-11.8%,12.3%]	0.965	-2.1% [-14.3%,10.2%]	0.730	+11.4% [2.0%,20.7%]	0.019
Immediate change in slope	+2.4% [1.1%,3.6%]	0.001	+1.8% [0.5%,3.1%]	0.010	+2.9% [2.0%,3.9%]	<0.001
Post-policy half-yearly trend	+1.1% [0.4%,1.8%]	0.003	+0.9% [0.2%,1.5%]	0.009	+0.6% [0.0%,1.2%]	0.061

Table A2.2: Early ANC initiation among users of 1+ ANC

	Early ANC (all women)		Early ANC (rural)		Early ANC (urban)	
	Estimate [95% CI]	p-value	Estimate [95% CI]	p-value	Estimate [95% CI]	p-value
Pre-policy starting level	14.0% [10.2%,17.9%]		10.7% [6.9%,14.6%]		23.0% [18.4%,27.6%]	
Pre-policy half-yearly trend	-0.3% [-0.9%,0.3%]	0.355	0.0% [-0.5%,0.6%]	0.899	-1.0% [-1.8%,-0.2%]	0.019
Immediate change in level	+2.6% [-2.5%,7.6%]	0.303	+0.1% [-4.6%,4.9%]	0.957	+6.0% [-3.0%,15.0%]	0.185
Immediate change in slope	+1.0% [0.3%,1.7%]	0.008	+0.4% [-0.2%,1.1%]	0.215	+2.1% [0.9%,3.2%]	0.001
Post-policy half-yearly trend	+0.7% [0.5%,0.9%]	<0.001	+0.4% [0.2%,0.7%]	0.001	+1.1% [0.4%,1.7%]	0.002

Table A2.3: Use of ANC from a public sector health facility among users of 1+ ANC

	Any public facility (all women)		Any public facility (rural)		Any public facility (urban)	
	Estimate [95% CI]	p-value	Estimate [95% CI]	p-value	Estimate [95% CI]	p-value
Pre-policy starting level	66.0% [59.6%,72.4%]		65.1% [56.5%,73.6%]		64.8% [58.4%,71.2%]	
Pre-policy half-yearly trend	+1.0% [0.0%,2.0%]	0.044	+1.2% [0.0%,2.5%]	0.050	+1.2% [0.1%,2.4%]	0.031
Immediate change in level	+3.0% [-6.1%,12.1%]	0.499	+2.6% [-8.1%,13.3%]	0.623	-4.1% [-16.7%,8.5%]	0.507
Immediate change in slope	-1.0% [-2.0%,0.0%]	0.042	-0.9% [-2.2%,0.3%]	0.129	-1.4% [-2.7%,-0.2%]	0.023
Post-policy half-yearly trend	0.0% [-0.2%,0.2%]	0.976	+0.3% [0.1%,0.5%]	0.007	-0.2% [-0.8%,0.4%]	0.502

APPENDIX 2: Study outcomes stratified by rural/urban residence

Table A2.4: Use of primary care facility among users of public facility-based ANC

	Primary care facility (all women)		Primary care facility (rural)		Primary care faciltiy (urban)	
	Estimate [95% CI]	p-value	Estimate [95% CI]	p-value	Estimate [95% CI]	p-value
Pre-policy starting level	64.5% [59.2%,69.8%]		69.4% [63.7%,75.1%]		49.0% [33.3%,64.7%]	
Pre-policy half-yearly trend	+0.4% [-0.5%,1.4%]	0.356	+0.4% [-0.5%,1.2%]	0.387	+0.1% [-2.2%,2.4%]	0.917
Immediate change in level	-4.7% [-14.6%, 5.2%]	0.335	-3.1% [-10.2,4.0%]	0.376	-13.0% [-36.4%,10.4%]	0.263
Immediate change in slope	-0.7% [-1.7%,0.4%]	0.193	-0.2% [-1.0%,0.6%]	0.645	+0.3% [-2.3%,2.9%]	0.800
Post-policy half-yearly trend	-0.2% [-0.7%,0.2%]	0.247	+0.2% [0.0%,0.3%]	0.042	+0.4% [-0.7%,1.6%]	0.440

Table A2.5: Received good content of care among users of public facility-based ANC

	Received all 6 routine ANC components (all women)		Received all 6 routine ANC components (rural)		Received all 6 routine ANC components (urban)	
	Estimate [95% CI]	p-value	Estimate [95% CI]	p-value	Estimate [95% CI]	p-value
Pre-policy starting level	9.4% [4.7%,14.2%]		7.5% [3.0%,12.0%]		13.4% [6.4%,20.4%]	
Pre-policy half-yearly trend	+0.4% [-0.6%,1.4%]	0.402	+0.4% [-0.5%,1.3%]	0.353	+0.7% [-0.8%,2.3%]	0.335
Immediate change in level	+4.9% [-4.9%,14.6%]	0.313	+3.9% [-4.6%,12.5%]	0.350	+8.0% [-9.2%,25.3%]	0.347
Immediate change in slope	+1.1% [-0.2%,2.3%]	0.087	+0.8% [-0.3%,1.9%]	0.162	+0.7% [-0.8%,2.1%]	0.337
Post-policy half-yearly trend	+1.5% [1.0%,2.0%]	<0.001	+1.2% [0.7%,1.7%]	<0.001	+1.4% [1.0%,1.9%]	<0.001

APPENDIX 2: Study outcomes stratified by rural/urban residence

Table A2.6: Summary of the impact of the 10/20 policy on ANC

	**Immediate change in level**	**Immediate change in slope**
**(2) 4+ ANC (most recent births)**		
All women	none	increased
Rural	none	increased
Urban	increased	increased
**(3) Early ANC (users of 1+ ANC)**		
All women	none	increased
Rural	none	none
Urban	none	increased
**(4) Public facility-based ANC (users of 1+ ANC)**		
All women	none	decreased
Rural	none	none
Urban	none	decreased
**(5) Public primary care facility (users of any public facility-based care)**		
All women	none	none
Rural	none	none
Urban	none	none
**(6) Received good content of ANC (users of any public facility-based care)**		
All women	none	none
Rural	none	none
Urban	none	none

increased: increasing effect or trend, $p<0.05$

decreased: decreasing effect or trend, $p<0.05$

none: no effect, $p>0.10$
