# Supplementary material for: Examining user fee reductions in public primary healthcare facilities in Kenya, 1997–2012: effects on the use and content of antenatal care
Source: Int J Equity Health. 2020 Mar 14;19:35. doi: 10.1186/s12939-020-1150-8 (PMC7073011; doi:10.1186/s12939-020-1150-8)
Supplement: Supplementary file 3 — Additional file 3. Mean gestational age calculations. [file 12939_2020_1150_MOESM3_ESM.pdf]

ADDITIONAL FILE 3: MEAN GESTATIONAL AGE CALCULATIONS

(1)%Births that are pre-term in sub-Saharan Africa*

12.30%

(2) Distribution of pre-term births by gestational age in sub-Saharan Africa*

		**Gestational age**		
	**All preterm births**	**<28 weeks**	**28 to <32 weeks**	**32 to <37 weeks**
**No. births**	3,933,200	204,700	409,500	3,319,000
**%pre-term births**	100.0%	5.2%	10.4%	84.4%

(3) Calculating median gestational age in sub-Sahararan Africa

	**Gestational age range**	**(A) %births occuring during gestational age range**	**(B) Median gestational age**	**(A * B) weight X median**
**full-term** 87.7% of all births	37 to <42 weeks	87.7%	39	34.2
**pre-term** 12.3% of all births	32 to <37 weeks	10.4%	34	3.5
	28 to <32 weeks	1.3%	29.5	0.4
	22 to <28 weeks	0.6%	24.5	0.2
			**weighted median gestational age**	**38.3**

**Estimates from: Blencowe H, Cousens S, Chou D, et al. Born Too Soon: The global epidemiology of 15 million preterm births. Reprod Health 2013;10:1–14. doi:10.1186/1742-4755-10-S1-S2*
