# Supplementary material for: Examining user fee reductions in public primary healthcare facilities in Kenya, 1997–2012: effects on the use and content of antenatal care
Source: Int J Equity Health. 2020 Mar 14;19:35. doi: 10.1186/s12939-020-1150-8 (PMC7073011; doi:10.1186/s12939-020-1150-8)

APPENDIX 4: Graphs of trends in ANC frequency, timing, source of care, and content of care

The figures on the following pages are graphical depictions of the results presented in tables 1-6 in the manuscript. The x-axis represents half-year periods; “h1” represents the first half of the year (January-June) and “h2” represents the second half of the year (July-December). The lines represent the predicted trend over time in coverage of the outcome variable. The circles represent the estimated coverage at each point; the size of each circle is proportional to the inverse of the variance for the estimated coverage during that half-year period.

APPENDIX 4: Graphs of trends in ANC frequency, timing, source of care, and content of care

Figure A5.1(a-c): Use of 4+ ANC among most recent births

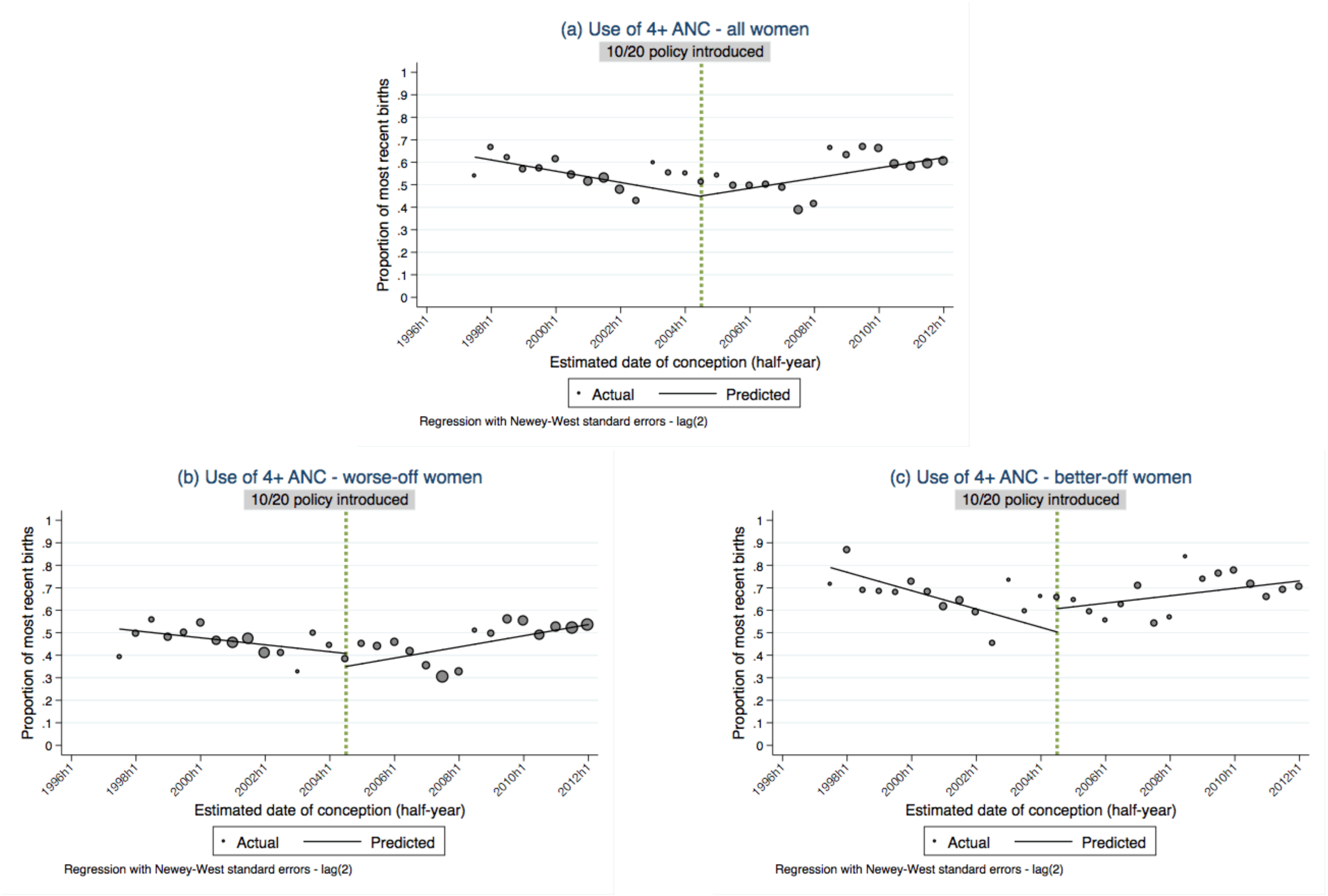

APPENDIX 4: Graphs of trends in ANC frequency, timing, source of care, and content of care

Figure A5.2(a-c): Early ANC initiation among users of 1+ ANC

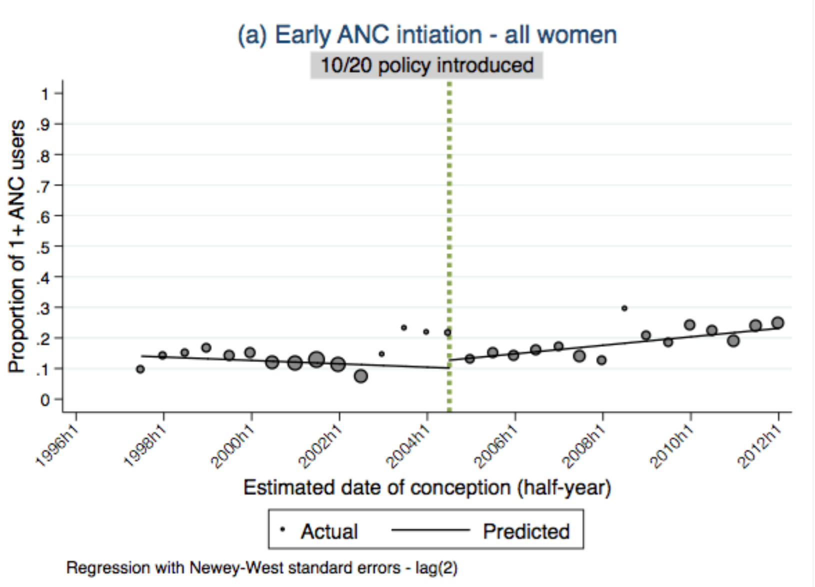

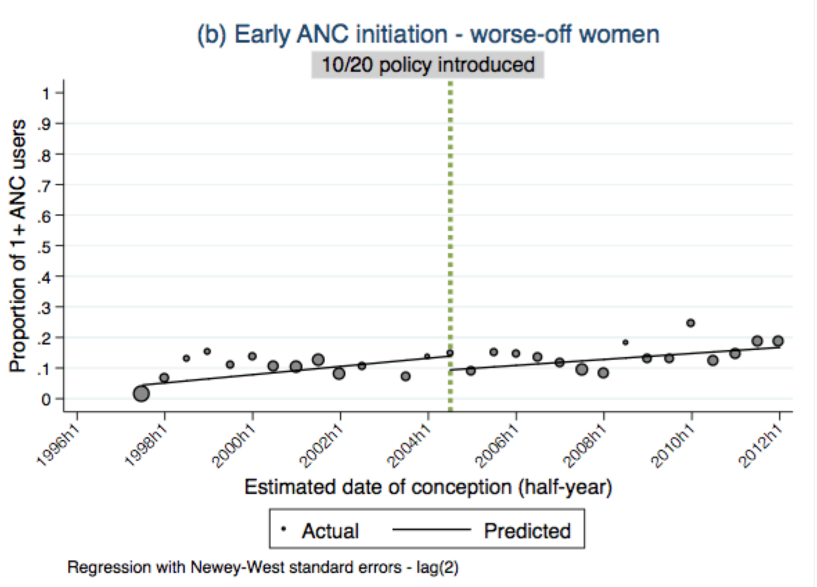

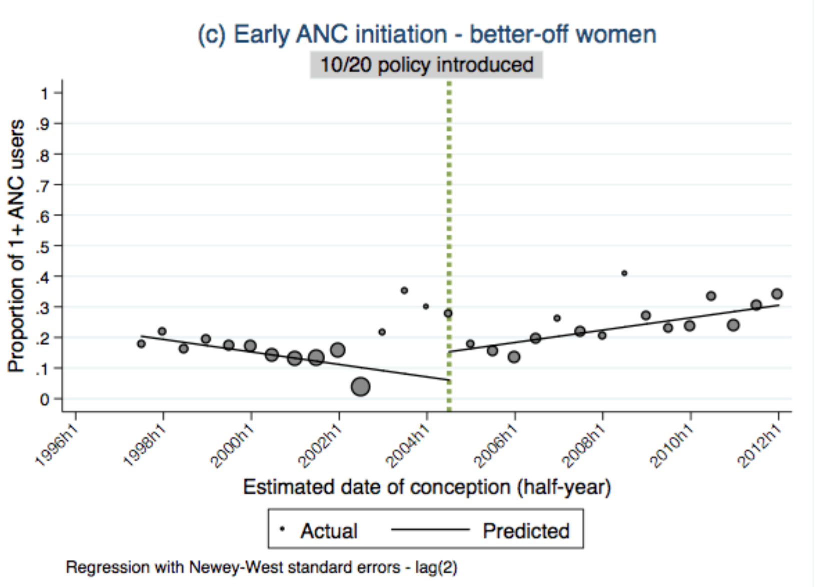

APPENDIX 4: Graphs of trends in ANC frequency, timing, source of care, and content of care

Figure A5.3(a-c): Use of ANC from a public sector health facility

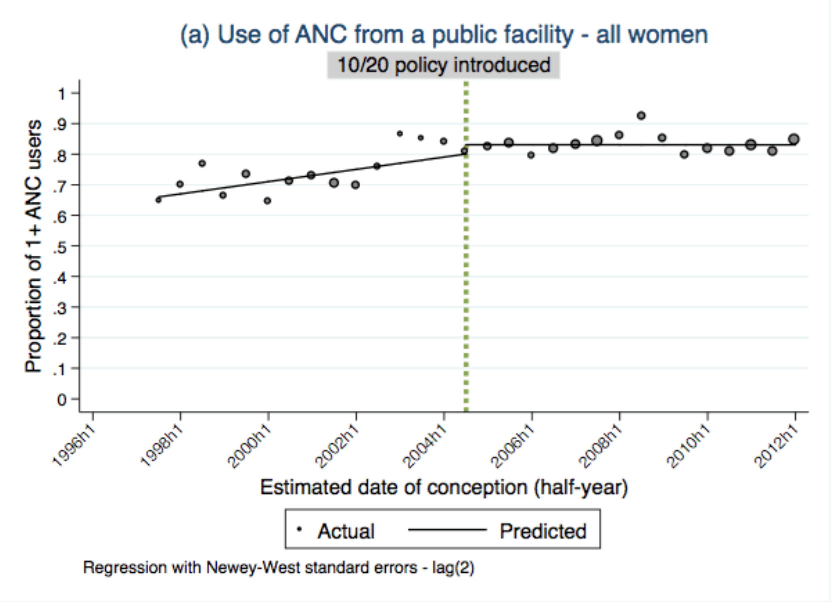

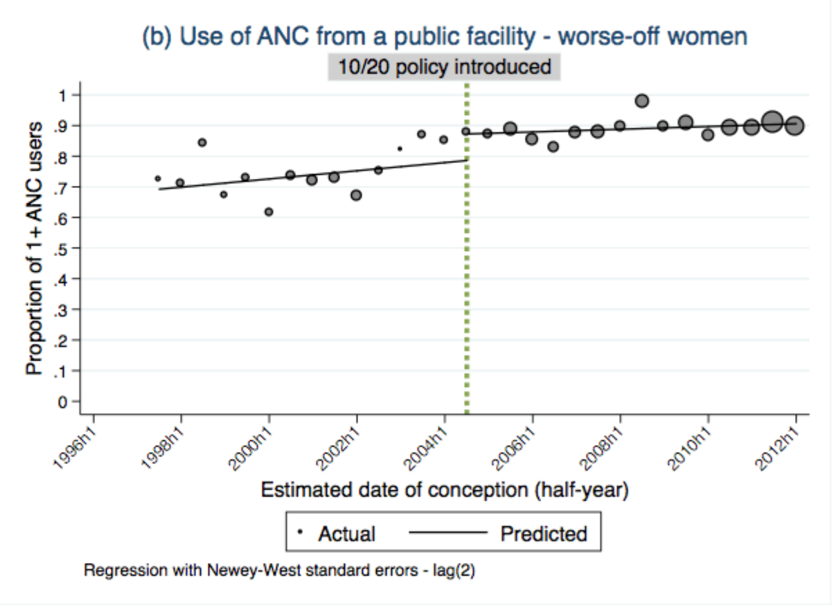

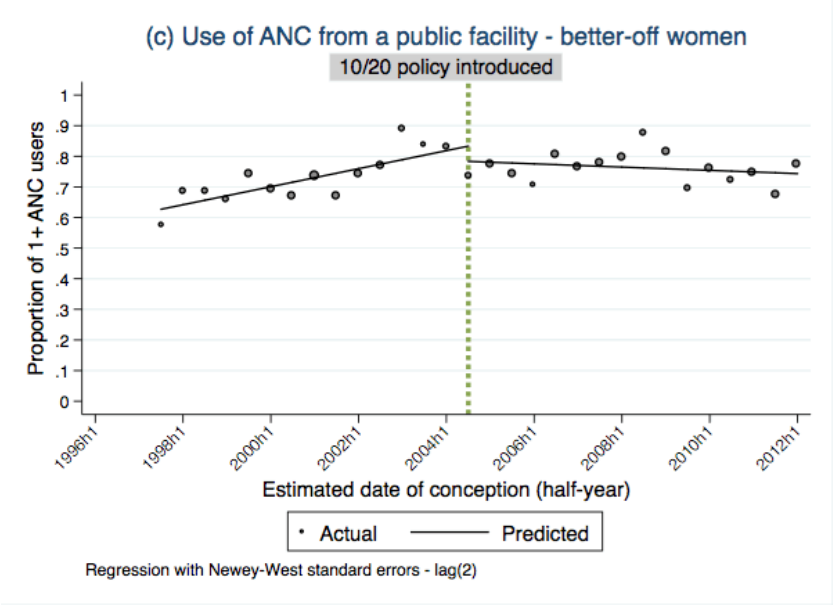

APPENDIX 4: Graphs of trends in ANC frequency, timing, source of care, and content of care

Figure A5.4(a-c): Use of primary care facilities among users of public facility ANC

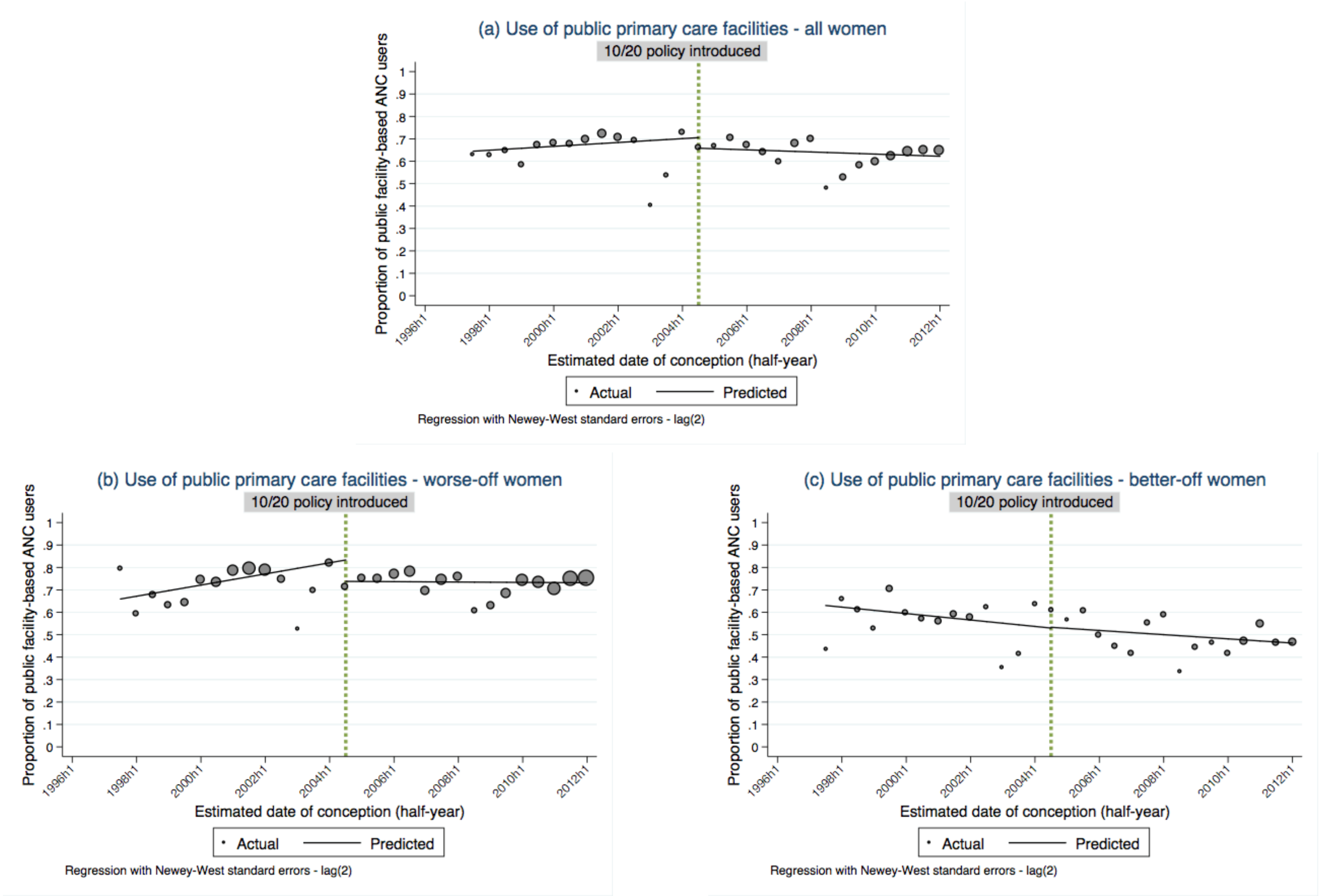

APPENDIX 4: Graphs of trends in ANC frequency, timing, source of care, and content of care

Figure A5.5(a-c): Received good content of care among users of public facility-based ANC

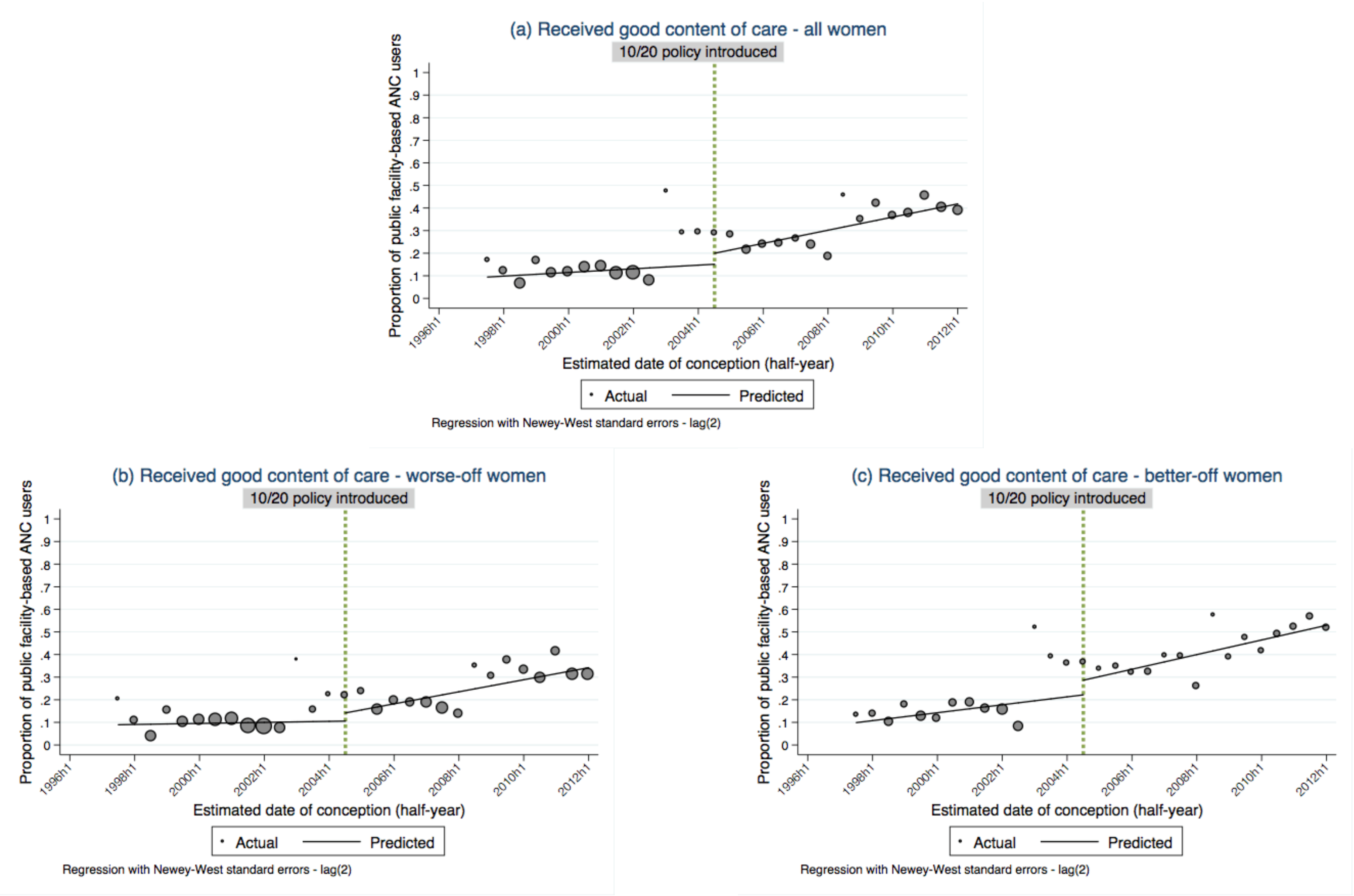

Supplement: Supplementary file 4 — Additional file 4. Graphs of trends in ANC frequency, timing, source of care, and content of care. [file 12939_2020_1150_MOESM4_ESM.pdf]
