# Supplementary material for: Examining user fee reductions in public primary healthcare facilities in Kenya, 1997–2012: effects on the use and content of antenatal care
Source: Int J Equity Health. 2020 Mar 14;19:35. doi: 10.1186/s12939-020-1150-8 (PMC7073011; doi:10.1186/s12939-020-1150-8)
Supplement: Supplementary file 5 — Additional file 5. Receipt of individual ANC components. [file 12939_2020_1150_MOESM5_ESM.pdf]

APPENDIX 5

Table A5.1: Receipt of six routine ANC components by facility type before and after introduction of the 10/20 policy

	Blood pressure measured	Urine sample taken	Blood sample taken	Received tetanus injection	Given iron supplements	Told about pregnancy complications	Received all six components
**Public hospital**							
Before 10/20	91.0%	71.6%	80.5%	96.7%	50.2%	45.9%	21.5%
After 10/20	94.1%	87.9%	94.3%	96.0%	74.0%	56.5%	39.5%
**Public health center**							
Before 10/20	80.6%	46.6%	56.7%	96.1%	48.2%	36.1%	11.6%
After 10/20	87.6%	78.6%	91.0%	94.2%	72.6%	51.4%	33.9%
**Public dispensary**							
Before 10/20	76.6%	37.6%	44.7%	95.0%	54.2%	27.6%	9.7%
After 10/20	86.0%	69.5%	85.8%	95.0%	71.2%	44.5%	25.8%
**Private facility**							
Before 10/20	89.9%	54.1%	58.9%	94.0%	57.3%	39.2%	13.4%
After 10/20	95.3%	85.4%	91.8%	95.0%	74.9%	57.9%	41.6%
**Home/other location**							
Before 10/20	32.4%	22.2%	25.9%	41.3%	22.7%	33.3%	7.5%
After 10/20	59.5%	32.3%	48.6%	67.5%	48.9%	27.2%	11.6%

Table A5.2: Receipt of ANC components by number of visits before and after the introduction of the 10/20 policy

	Blood pressure measured	Urine sample taken	Blood sample taken	Received tetanus injection	Given iron supplements	Told about pregnancy complications	Received all six components
**1-3 visits**							
Before 10/20	77.1%	42.5%	49.5%	92.3%	48.9%	31.2%	9.6%
After 10/20	85.1%	70.1%	85.2%	92.7%	67.5%	41.8%	24.5%
**4-7 visits**							
Before 10/20	86.9%	56.3%	64.8%	95.8%	52.6%	39.8%	14.9%
After 10/20	93.3%	85.4%	93.6%	96.2%	76.1%	58.5%	40.5%
**8+ visits**							
Before 10/20	93.0%	68.8%	76.8%	94.0%	58.5%	51.9%	24.9%
After 10/20	95.0%	95.5%	96.3%	96.4%	76.3%	65.2%	49.6%
